# Effects of Spearfishing on Reef Fish Populations in a Multi-Use Conservation Area

**DOI:** 10.1371/journal.pone.0051938

**Published:** 2012-12-12

**Authors:** Ashley J. Frisch, Andrew J. Cole, Jean-Paul A. Hobbs, Justin R. Rizzari, Katherine P. Munkres

**Affiliations:** 1 ARC Centre of Excellence for Coral Reef Studies, James Cook University, Townsville, Australia; 2 School of Marine and Tropical Biology, James Cook University, Townsville, Australia; 3 Oceans Institute and School of Plant Biology, The University of Western Australia, Perth, Australia; Leibniz Center for Tropical Marine Ecology, Germany

## Abstract

Although spearfishing is a popular method of capturing fish, its ecological effects on fish populations are poorly understood, which makes it difficult to assess the legitimacy and desirability of spearfishing in multi-use marine reserves. Recent management changes within the Great Barrier Reef Marine Park (GBRMP) fortuitously created a unique scenario by which to quantify the effects of spearfishing on fish populations. As such, we employed underwater visual surveys and a before-after-control-impact experimental design to investigate the effects of spearfishing on the density and size structure of target and non-target fishes in a multi-use conservation park zone (CPZ) within the GBRMP. Three years after spearfishing was first allowed in the CPZ, there was a 54% reduction in density and a 27% reduction in mean size of coral trout (*Plectropomus* spp.), the primary target species. These changes were attributed to spearfishing because benthic habitat characteristics and the density of non-target fishes were stable through time, and the density and mean size of coral trout in a nearby control zone (where spearfishing was prohibited) remained unchanged. We conclude that spearfishing, like other forms of fishing, can have rapid and substantial negative effects on target fish populations. Careful management of spearfishing is therefore needed to ensure that conservation obligations are achieved and that fishery resources are harvested sustainably. This is particularly important both for the GBRMP, due to its extraordinarily high conservation value and world heritage status, and for tropical island nations where people depend on spearfishing for food and income. To minimize the effects of spearfishing on target species and to enhance protection of functionally important fishes (herbivores), we recommend that fishery managers adjust output controls such as size- and catch-limits, rather than prohibit spearfishing altogether. This will preserve the cultural and social importance of spearfishing in coastal communities where it is practised.

## Introduction

Overfishing has been identified as one of the greatest threats to the future of coral reefs [1], [2]. Understanding and managing the ecological effects of reef fisheries is therefore crucial for conserving coral reefs, and for bringing wealth and stability to the millions of people who depend on coral reefs for food or income. An important step in this direction is to understand how various fishing methods impact upon both target and non-target organisms.

A wide variety of fishing gears such as spearguns, traps and handlines are used in reef fisheries throughout the world. Each gear type selects for particular sizes and species of fishes, and has a characteristic efficiency (i.e. catch-per-unit-effort; CPUE) and collateral impact (i.e. bycatch and pollution) [3], [4], [5]. Accordingly, each gear type is likely to affect reef resources in fundamentally different ways and at different rates. In some acute cases, this may manifest at the ecosystem level [6], [7], because different gear types target different functional groups of fishes (e.g. herbivores versus piscivores). Prudent fishery managers should consider these differences and promote gear types that minimize the negative effects of fishing on functionally important fish populations and associated ecosystems. This type of adaptive management is imperative for enhancing the resilience of coral reefs to intensifying anthropogenic stressors such as climate change [5].

Spearfishing is one of the most common, yet controversial, forms of fishing on coral reefs. It is highly selective, both in terms of species and size [3] and thus has minimal direct impact on non-target species [4]. Additionally, breath-hold spearfishing is limited to shallow water, so the proportion of target fishes available to spearfishers is typically less than the proportion available to users of other gear types such as traps and lines. Nevertheless, spearfishing is often perceived to be more efficient (in terms of CPUE) and thus more destructive to fish populations than alternative gear types [8], [9]. Spearfishing also allows the targeting of keystone species such as herbivorous parrotfishes, which have critical ecosystem functions in maintaining reefs in a coral dominated state [5], [6]. For these reasons, the legitimacy and desirability of spearfishing have often been questioned, and appeals for stringent regulation or prohibition have emerged in developed and developing countries [5], [8], [10].

Despite the global popularity of spearfishing, information pertaining to its effects and yields is scarce. Limited catch and effort data are available for a few tropical reef spearfisheries [4], [9], but the effects of spearfishing on reef fish populations are yet to be investigated. This dearth of information about spearfishing has resulted in a distinct lack of management action in many countries where spearfishing is prevalent [8], [11], which increases the risk of over-fishing and associated flow-on effects such as phase-shifts in habitat structure [12]. To assist policy makers to implement sound knowledge-based management strategies, detailed assessments of the effects of spearfishing on reef fish populations are urgently needed.

Marine reserves have been widely advocated as an effective means of managing multi-species reef fisheries; they conserve biodiversity, enhance fisheries in outlying regions, and may act as reference areas to quantify the effects of human activities such as fishing [13], [14]. The Great Barrier Reef Marine Park (GBRMP), Australia, is a large multi-use marine reserve that is managed using a system of spatial zoning, introduced progressively between 1981 and 1988 [15]. Within each zone, certain activities are allowed whilst others are prohibited, thereby creating a network of fished and unfished areas. In 2004, the GBRMP was rezoned, which altered the spatial distribution of sanctioned activities in some zones. In particular, spearfishing became allowed in some multi-use Conservation Park Zones (CPZ) where it was previously prohibited. This unique scenario created conditions suitable for quantifying the effects of spearfishing on reef fish populations. The objective of our study, therefore, was to employ a before-after-control-impact (BACI) experimental design to quantify the effects of spearfishing on the density and size structure of target and non-target fishes on shallow coral reefs of the GBRMP. Consistent with conventional wisdom, we hypothesized that accession of spearfishing to a CPZ would reduce the density and size structure of target species, without equivalent changes at nearby ‘control’ zones where spearfishing remained prohibited. Importantly, this type of experimental approach should provide the necessary evidence to inform policy debate on the legitimacy and desirability of spearfishing, both in Australia and in other tropical maritime nations where spearfishing occurs.

## Materials and Methods

### Study Site and Fishery

**Figure 1:**
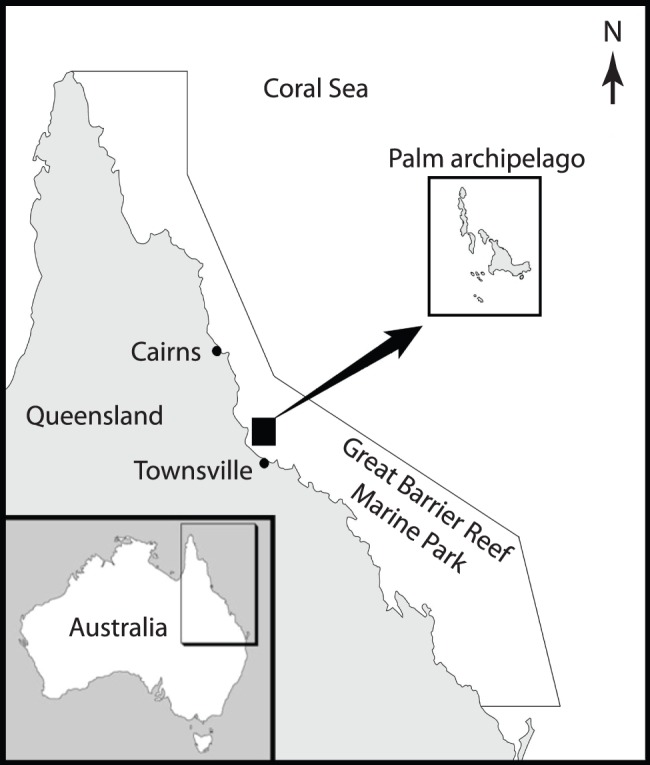
Map of the Palm archipelago in the Great Barrier Reef Marine Park, Australia.

The study was conducted at Orpheus and Palm Islands in the central section of the GBRMP (Figure 1; GBRMP permit no. G05/15590.1). Both islands have well-developed fringing reefs comprised of a high diversity and moderate coverage of hard and soft corals. Generally, there is a well-defined reef flat (1–3 m deep) and a steeply descending reef slope (3–20 m deep), beyond which the substrate is flat and sandy. Unlike the reef flat, the reef slope has high topographic complexity with numerous ledges and coral-covered outcrops (locally known as bommies). Fish diversity and abundance, and hence fishing effort, are concentrated on the reef slope. Because of the broadly equivalent reef morphology and hydrodynamic environment across the Palm archipelago, Orpheus and Palm Islands are ideal locations for paired comparisons of fish populations (i.e. with and without spearfishing).

Palm Island is inhabited (*c*. 3000 people) and close to major urban centres on the mainland (e.g. Townsville). The Palm archipelago is therefore a relatively high-use area for recreational and indigenous fishers, and fishing pressure is regarded as moderate to high by Australian standards [16], [17]. Linefishing and spearfishing (without SCUBA) are the most popular forms of reef fishing, although the effort expended in each activity is not currently known. All regulations that apply to linefishing (e.g. spatial closures, catch limits) also apply to spearfishing, but some additional spatial closures apply only to spearfishing (see below). Fishing regulations are well enforced by government agencies and the level of non-compliance is relatively low [16]. This suggests that the fish community in the no-fishing zone at Orpheus Island is intact and thus provides a suitable ecological baseline.

Although the diversity of food fishes in the region is very large, spearfishers typically have strong preferences for a few select species, some of which are also sought by linefishers (Table 1). The primary target species of both spearfishers and linefishers is coral trout (*Plectropomus* spp.), which are large, long-lived groupers (Family Serranidae) [19], [20]. With respect to behavior, coral trout are non-schooling, conspicuous and relatively sedentary, which makes them easy to quantify by underwater visual census (UVC) and highly susceptible to spearfishing. Species other than coral trout are not generally targeted by spearfishers, but some are captured opportunistically. For example, parrotfish (*Scarus* spp.) and stripey snapper (*Lutjanus carponotatus*), which are less desirable to eat and more difficult to spear than coral trout, typically comprise only 6% and 2% respectively of spearfisher’s catches [4]. Catch composition is also influenced to a small degree by legal size- and catch-limits (Table 1).

**Table 1:** Common species of reef fishes at the Palm archipelago and their importance to spearfishers and linefishers (based on data from [4]).

Taxa	Importance tospearfishing	Importance tolinefishing	Min. legal size(cm total length)	Catch limit(per person)^a^
Coral trout, *Plectropomus spp.*	Primary target species	Primary target species	38	7 (combined for all species)
Stripey snapper, *Lutjanus carponotatus*	Opportunistic only	Secondary target species	25	5
Parrotfish, *Scarus spp.*	Opportunistic only	Not captured	25	5 (per species)
Bommie cod, *Cephalopholis cyanostigma*	Not captured	Bycatch (discarded)	38^b^	5 (combined for all rock cods)^b^
Rabbitfish, *Siganus doliatus*	Not captured	Not captured	none	none

a In addition to individual catch limits for each taxa, there is a combined catch limit of 20 for all coral reef fish [18].

b Bommie cod, a bycatch species, have a minimum legal size and catch limit by default under Queensland State law [18].

### Experimental Design

**Figure 2:**
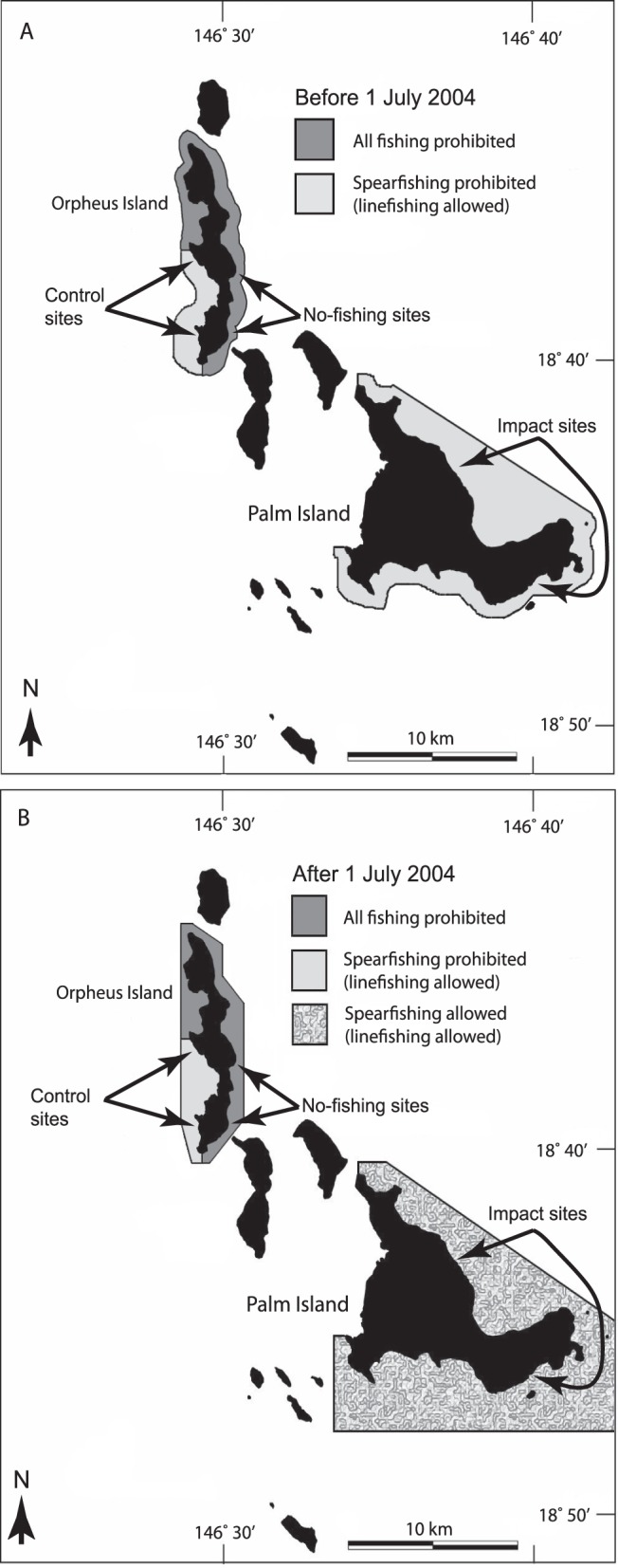
Maps showing the spatial arrangement of fishing zones before (A) and after (B) 1 July 2004. Arrows show the approximate locations of survey sites within ‘no-fishing’, ‘control’ and ‘impact’ zones. The no-fishing zone (north and east Orpheus Island) has been protected from all forms of fishing since 1987. The control zone (south-west Orpheus Island) has been protected from spearfishing since 1987. The impact zone (Palm Island) was protected from spearfishing from 1987 to 1 July 2004, but spearfishing was allowed from 1 July 2004 onwards. Linefishing was allowed at control and impact zones before and after 1 July 2004. For clarity, other multi-use management zones in the Palm archipelago are not shown (see www.gbrmpa.gov.au for further information).

Prior to 1 July 2004, spearfishing was prohibited in the CPZs at Palm and Orpheus Islands. However, from 1 July 2004, spearfishing was allowed in the CPZ at Palm Island, but spearfishing remained prohibited in the CPZ at Orpheus Island (Figure 2). Accordingly, fish populations were surveyed before (2004) and after (2005, 2007, 2009) accession of spearfishing to the CPZ at Palm Island (the ‘impact’ zone), with equivalent surveys undertaken in the CPZ at Orpheus Island (the ‘control’ zone). Any effects of spearfishing were identified by the presence of statistical interaction between the two sources of variation (zone × year). Although accession of spearfishing to CPZs occurred elsewhere in the GBRMP, it was not possible to replicate at the level of island group because Orpheus and Palm Islands were the only paired (juxtaposed) combination of control-impact CPZs available.

CPZs are multi-use zones and limited linefishing (one line and hook per person) was allowed in the CPZs at both Palm and Orpheus Islands before and after 1 July 2004. It was assumed that the spatial and temporal distribution of linefishing effort remained unchanged for the duration of this study and did not influence our evaluation of the effects of spearfishing. The validity of this assumption is strongly supported by four lines of evidence. Firstly, rezoning of the GBRMP had no effect on the area of fringing reef available for linefishing at either Palm or Orpheus Island, and had only a small effect (−3%) on the available reef area across the entire Palm archipelago. Secondly, boat-ramp surveys on the adjacent coast (Lucinda) found that linefishing effort at Orpheus and Palm Islands in 2004 and 2005 did not change significantly (χ^2^ test, χ^2^_1_ = 0.04, *p*>0.75, *N* = 227 fisher days, 36 participants). Thirdly, the number of boats observed by the authors while visiting Orpheus and Palm Islands in 2004, 2005 and 2007 (4 d per island per year) did not differ significantly between years (χ^2^ test, χ^2^_2_ = 2.05, *p*>0.25, *N* = 31 boats). Lastly, the quantity of lost fishing line observed at each of the study sites in 2004, 2005, 2007 and 2009 did not differ significantly between years (χ^2^ test, χ^2^_3_ = 2.80, *p*>0.25, *N* = 54 lines). Taken together, these four pieces of evidence strongly indicate that the spatial and temporal distribution of linefishing effort at Palm and Orpheus Islands remained unchanged for the duration of this study.

Although spearfishing effort in the impact zone was not quantified, it was assumed that spearfishers accessed the CPZ from 1 July 2004 onwards. Typically, fishers are very enthusiastic about opportunities to operate in previously closed areas (GBRMP Authority, pers. comm.). For example, when Bramble Reef (∼30 km north of Palm Island) was opened to fishing in 1995 after 3.5 yr of closure, there were 45 vessels and >90 fishers (commercial and recreational) present on opening day [21]. Therefore, spearfishing effort in the impact zone at Palm Island was likely to be substantial from July 2004 onwards.

Part of Orpheus Island has been a no-take marine reserve since 1987 and is designated here as a ‘no-fishing’ zone (Figure 2). Additional fish surveys were undertaken in this zone to evaluate natural variation in fish populations in the absence of both spearfishing and linefishing. The no-fishing zone was not used as a control for the BACI design because the absence of linefishing in this zone created potential inequalities with the impact zone, even before accession of spearfishing in 2004.

### Visual Surveys

Fish surveys consisted of standard UVC with strip-transects (6 m×50 m) as the unit of replication. The number and size of target and non-target fishes (as controls) were recorded by a single observer (A.J.F.) with the aid of SCUBA. Count data were standardized to units of density (fish per 1000 m^2^) and total length (TL) of coral trout and stripey snapper was estimated to the nearest 5 cm. Due to cryptic behaviour, small fish (<10 cm TL) may have been under-sampled by UVC. Indices of habitat quality were visually estimated in 2 m^2^ quadrats every 5 m by a second observer (A.J.C. or K.P.M.). These indices consisted of live hard coral cover (%), total live coral cover (%; includes soft coral) and structural complexity, which was estimated on a scale of one to five, as follows: 1 = flat, sandy and featureless; 2 = dominated by rubble, rocks, algae, encrusting corals, but highly planar with few refuges; 3 = abundant rocks and/or coral with limited three-dimensional structure, but occasional overhangs; 4 = well developed coral or rock structures with overhangs, but few large bommies and caves; 5 = multi-layered coral matrix with caves, canyons, large bommies and abundant overhangs.

Due to natural variability in the distribution of reef fishes across space and time, fish surveys were replicated at two sites (within zones), two months (April and June) and two depths (4–6 m and 8–15 m). Sampling occurred in April and June of each year (2004, 2005, 2007, 2009) because the new zoning plan for the GBRMP was not available until late March 2004 and was implemented on 1 July 2004. Hence, it was not possible to collect a long time-series of ‘before’ samples. The maximum depth for all surveys was 15 m because this was considered to be the maximum working depth of most spearfishers, since the use of SCUBA for spearfishing is prohibited in the GBRMP. Five surveys were replicated at each combination of zone, year, month, site and depth, making a total of 320 surveys, plus an additional 160 surveys in the no-fishing zone. The position of each transect was chosen haphazardly during each and every sampling occasion, and consecutive transects were well-spaced (nominally 30 m) to ensure independence.

Cyclone Hamish hit the Queensland coast in March 2009, just before the field surveys in April and June of that year. Fish counts (especially coral trout) were lower than normal, apparently because of the physical disturbance caused by the cyclone. Elsewhere in the GBRMP, commercial catch-rates (linefishing) of coral trout declined by 30% in the 9 mo following the cyclone [22]. Given that declines in fish abundance are not uncommon after severe storms [23], [24], data collected in 2009 were interpreted with caution or excluded (see below).

### Statistical Analyses

Fish count data contained many zero estimates and often did not satisfy the assumptions of parametric statistical tests. Thus, data were pooled across sites, months and depths. To test for effects of spearfishing, density data from control and impact zones were analyzed by two-factor analysis of covariance (ANCOVA), with total coral cover, hard coral cover and structural complexity as the covariates. These habitat indices were also analyzed as stand-alone variables using standard analysis of variance (ANOVA). To test for significant temporal variation that was independent of fishing, density data from the no-fishing zone were analyzed by one-factor ANCOVA using the same covariates as above. If there was a significant difference in fish density between years within the no-fishing zone, then BACI data were re-analyzed without 2009 data (to eliminate potential bias caused by Cyclone Hamish). In separate analyses, densities of ‘legal-sized’ coral trout and stripey snapper in control and impact zones were analyzed by non-parametric Kruskal-Wallace tests, since the data failed to meet parametric assumptions. Coral trout and stripey snapper were considered to be of legal size if their estimated TL was ≥40 and ≥25 cm, respectively. As before, if a significant difference in fish density existed between years within the no-fishing zone, then 2009 data were excluded from the BACI analyses.

For coral trout and stripey snapper, size data were analyzed by one-factor ANOVA (no-fishing zone) or two-factor ANOVA (BACI). In separate analyses for each zone, size distributions of coral trout and stripey snapper were analyzed by χ^2^ homogeneity tests. Each size distribution was analyzed twice, first using a broad range of size categories (*k* = 5 after pooling) and second using only two size categories (<minimum legal size and ≥minimum legal size) to remove the disproportionate influence of numerous ‘under-size’ categories. For each pair of tests, Bonferroni’s adjustment was applied to prevent inflation of Type I error rate (adjusted α = 0.025).

For each parametric statistical test, the relevant assumptions were checked *a priori* using probability plots (for normality) and Levene’s test (for homogeneity of variance). Heteroscedastic data were transformed (*y* = Log_10_[*x*+1]) or analyzed using non-parametric methods. Where possible, group means were compared using Tukey’s HSD *post hoc* test. All statistical analyses were performed using SPSS computer software (SPSS, Chicago, U.S.A.) and a significant difference was considered to exist if *p*<0.05, unless otherwise stated. All data in the text and figures are presented as the arithmetic mean of untransformed data (± one standard error, SE).

## Results

### Density of Target Fishes

**Figure 3:**
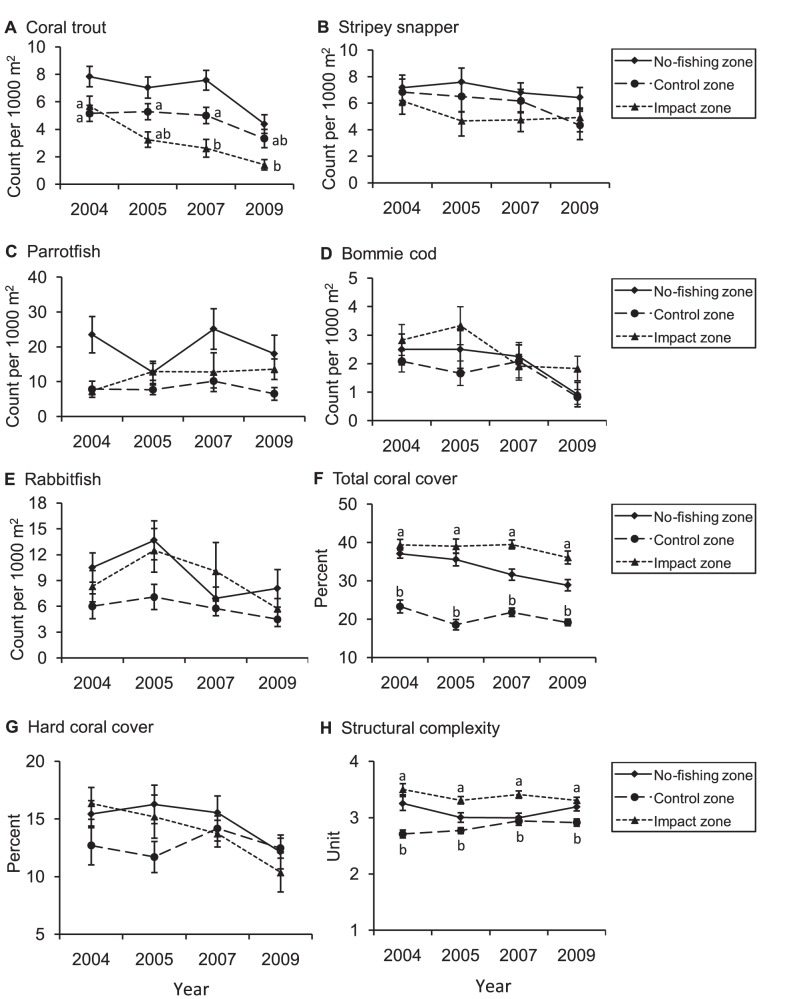
Mean fish density (A-E), coral cover (F, G) and structural complexity (H) (±1SE) from 2004 to 2009. Data for the no-fishing zone are shown only for the purpose of comparison. The management history of each zone is described in Figure 2. Structural complexity was defined on a scale of zero to five (see text for definitions). Means were calculated from 40 samples (underwater visual transects) per zone per year. Groups with the same letters were not significantly different.

Mean densities of coral trout, stripey snapper and parrotfish in the no-fishing zone were generally higher than those in fished (control and impact) zones and were relatively stable through time, except that the density of coral trout declined significantly in 2009, presumably as a result of Cyclone Hamish (Figure 3, Table 2). Hence, coral trout density data for 2009 were excluded from subsequent BACI analyses.

**Table 2:** Results of one-factor ANCOVA of fish density in the no-fishing zone, with habitat indices as covariates^a^.

Source of variation	Total coral cover (d.f. = 1, 153)	Hard coral cover (d.f. = 1, 153)	Structural complexity (d.f. = 1, 153)	Year (d.f. = 3, 153)
Coral trout	0.641	0.173	0.576	0.012
Stripey snapper	0.677	0.233	0.085	0.893
Parrotfish^b^	0.008	0.331	0.082	0.447
Bommie cod	0.009	0.001	0.215	0.090
Rabbitfish	0.498	0.557	0.568	0.102
Hard coral cover	–	–	–	0.191^c^
Total coral cover	–	–	–	0.037^c^
Complexity^b^	–	–	–	0.064^c^

a Also shown are results of one-factor ANOVA of habitat indices. Data originate from 40 underwater visual transects per year (2004, 2005, 2007 and 2009). Values are probabilities. d.f., degrees of freedom.

b Data were transformed (*y* = Log_10_[*x*+1]) to homogenize variances.

c d.f. = 3, 156.

Between 2004 and 2007, mean density of coral trout in the control zone was relatively stable (range: 5.00–5.25 fish per 1000 m^2^), but mean density of coral trout in the impact zone declined by 54% (from 5.63±0.72 to 2.58±0.65 fish per 1000 m^2^; Figure 3A). Statistical interaction between zone and year was significant (Table 3), indicating that the reduced density of coral trout at Palm Island was an effect of spearfishing. In contrast, spearfishing had no apparent effect on the densities of stripey snapper or parrotfish (Figure 3B, 3C; Table 3). These trends in fish density remained unchanged when analyses were limited to legal-size fish only (Table 4). In particular, mean density of legal-size coral trout in the no-fishing zone declined significantly in 2009, presumably as a result of Cyclone Hamish (Table 4). Between 2004 and 2007, mean density of legal-size coral trout in the control zone was relatively stable (range: 2.03–2.12 fish per 1000 m^2^), but mean density of legal-size coral trout in the impact zone declined significantly (i.e. by 67%, from 1.93±0.15 fish per 1000 m^2^ in 2004 to 0.64±0.06 fish per 1000 m^2^ in 2007). No significant differences in density of legal-size stripey snapper were detected between years in either the no-fishing, control or impact zones (Table 4).

**Table 3:** Results of two-factor ANCOVA comparing fish density between zones (control versus impact) and between years (2004–2009), with habitat indices as covariates^a^.

Source of variation	Total coral cover (d.f. = 1, 309)	Hard coral cover (d.f. = 1, 309)	Structural complexity (d.f. = 1, 309)	Zone(d.f. = 1, 309)	Year(d.f. = 3, 309)	Zone × Year(d.f. = 3, 309)
Coral trout^b^	0.378	0.286	0.613	0.048	0.001	0.118
Coral trout (excl. 2009 data)	0.168^c^	0.190^c^	0.773^c^	0.286^c^	0.038^d^	0.043^d^
Stripey snapper^b^	0.958	0.818	0.013	0.445	0.100	0.299
Parrotfish^b^	0.437	0.773	0.164	0.362	0.363	0.214
Bommie cod^b^	0.153	0.050	0.305	0.225	0.046	0.238
Rabbitfish	0.182	0.321	0.916	0.010	0.111	0.669
Hard coral cover	–	–	–	0.254^e^	0.145^f^	0.107^f^
Total coral cover	–	–	–	0.001^e^	0.305^f^	0.755^f^
Complexity	–	–	–	0.001^e^	0.291^f^	0.280^f^

a Also shown are results of two-factor ANOVA of habitat indices. Data originate from 40 underwater visual transects per zone per year. Values are probabilities. d.f., degrees of freedom.

b Data were transformed (*y* = Log_10_[*x*+1]) to homogenize variances.

c d.f. = 1, 231.

d d.f. = 2, 231.

e d.f. = 1, 312.

f d.f. = 3, 312.

**Table 4:** Results of Kruskal-Wallace tests comparing the density of legal-size coral trout and stripey snapper between years^a^.

Source of variation	Zone	Data range	χ^2^ statistic	Degrees of freedom	Probability
Coral trout	No-fishing	2004–2009	10.43	3	0.015
	Control	2004–2007	1.81	2	0.404
	Impact	2004–2007	6.76	2	0.034
Stripey snapper	No-fishing	2004–2009	3.18	3	0.365
	Control	2004–2009	7.77	3	0.051
	Impact	2004–2009	6.43	3	0.092

a Coral trout and stripey snapper were considered to be of legal size if their estimated TL was ≥40 and ≥25 cm, respectively. As there was a significant difference in fish density between years within the no-fishing zone, the data for 2009 were excluded from BACI analyses (to remove potential bias caused by Cyclone Hamish).

### Density of Non-target Fishes

Mean density of bommie cod (*Cephalopholis cyanostigma*) in the no-fishing zone appeared to decline in 2009 (Figure 3D), but this result was not statistically significant (Table 2). For the BACI analyses, mean density of bommie cod was significantly different between years, but there was no significant interaction (zone×year; Table 3), indicating that temporal changes in density were unrelated to spearfishing. Mean density of rabbitfish (*Siganus doliatus*) was significantly lower in the control zone relative to the impact zone, but there was no significant interaction between year and zone (Figure 3E, Table 3).

### Size of Target Fishes

**Figure 4:**
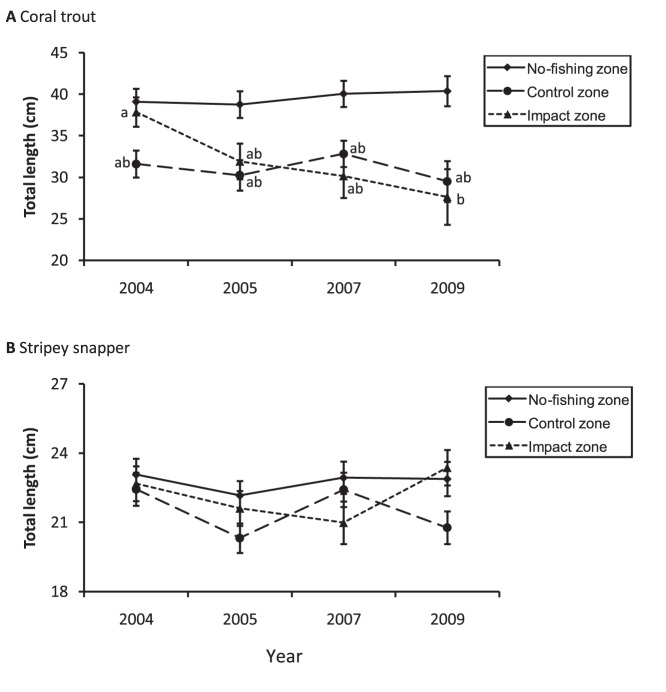
Mean total length (±1SE) of coral trout (A) and stripey snapper (B) from 2004 to 2009. Data for the no-fishing zone are shown only for the purpose of comparison. The management history of each zone is described in Figure 2. Sample sizes for control, impact and no-fishing zones were 40–62, 17–68 and 53–94 respectively. Data originate from 40 underwater visual transects per zone per year. Groups with the same letters were not significantly different.

Mean sizes (TL) of coral trout and stripey snapper in the no-fishing zone were generally larger than those in fished (control and impact) zones and were relatively stable through time (range: 38.76–40.38 cm TL for coral trout and 22.17–23.07 cm TL for stripey snapper), despite the presence of Cyclone Hamish in 2009 (Figure 4). Mean size of coral trout in the control zone was also relatively stable through time (range: 29.50–32.83 cm TL), but mean size of coral trout in the impact zone declined significantly (i.e. by 27%, from 37.87±1.76 cm TL in 2004 to 27.65±3.35 cm TL in 2009). Statistical interaction between zone and year was significant (Table 5), indicating that the reduced size of coral trout at Palm Island was an effect of spearfishing. No significant differences in mean size of stripey snapper were detected between years in either the no-fishing, control or impact zones (Table 5).

**Table 5:** Results of two-factor ANOVA comparing fish length between zones (control and impact) and between years (2004–2009)^a^.

Source of variation	Zone	Year	Zone × Year
Coral trout (control *v*.impact)	0.588(1, 372)	0.045 (3, 372)	0. 032 (3, 372)
Coral trout (no-fishing)	–	0.897 (3, 319)	–
Stripey snapper (control *v*. impact)	0.212(1, 525)	0.171 (3, 525)	0.073 (3, 525)
Stripey snapper (no-fishing)	–	0.848 (3, 329)	–

a Also shown are results of one-factor ANOVA of fish length in the no-fishing zone between years (2004–2009). Data originate from 40 underwater visual transects per zone per year. Values are probabilities with degrees of freedom in parentheses.

**Figure 5:**
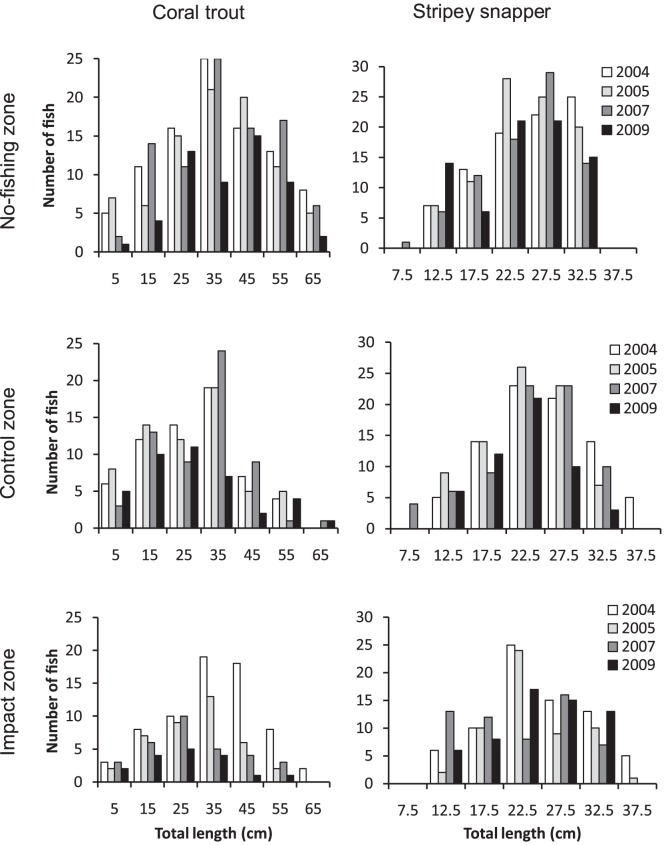
Length-frequency distributions of coral trout and stripey snapper. Data originate from 40 underwater visual transects per zone per year. The management history of each zone is described in Figure 2. The *x*-axis labels are size-category midpoints. Minimum legal sizes of coral trout and stripey snapper are 38 and 25 cm total length respectively.

The size structure of coral trout in the no-fishing zone (all years) was broadly similar to a standard normal curve (Figure 5). In contrast, the size structures of coral trout in control and impact zones (all years) appeared truncated, with relatively few individuals ≥40 cm TL, which approximates the minimum legal size of this species group (Figure 5). In 2004 (before spearfishing was allowed), 41% of coral trout in the impact zone were ≥40 cm TL. After spearfishing was allowed, the proportion declined to 21% in 2005 and only 12% in 2009. These temporal differences were statistically significant and were related to spearfishing, since there were no significant temporal differences in the proportion of legal-size versus under-size coral trout in the control zone (Table 6).

**Table 6:** Results of χ^2^ homogeneity tests comparing size distributions of coral trout and stripey snapper in no-fishing, control and impact zones between years (2004–2009)^a^.

Source of variation	Zone	No. of size categories (d.f.)^b^	χ^2^ statistic	Probability^c^
Coral trout	No-fishing	5 (12)	10.21	0.598
		2 (3)	1.41	0.703
	Control	5 (12)	13.02	0.368
		2 (3)	0.25	0.969
	Impact	5 (12)	15.76	0.203
		2 (3)	9.50	0.023
Stripey snapper	No-fishing	5 (12)	14.53	0.268
		2 (3)	1.40	0.706
	Control	5 (12)	16.99	0.150
		2 (3)	7.61	0.055
	Impact	5 (12)	25.33	0.013
		2 (3)	1.87	0.599

a Data originate from 40 underwater visual transects per zone per year.

b Each size distribution was analyzed twice, first using a broad range of size categories (*k* = 5 after pooling) and second using only two size categories (<minimum legal size and ≥minimum legal size). d.f., degrees of freedom.

c A significant difference was considered to exist if *p*<0.025, as per Bonferroni’s adjustment.

The size structure of stripey snapper in no-fishing and control zones was not significantly different among years (Figure 5, Table 6). In contrast, the size structure of stripey snapper in the impact zone was significantly different among years, but only when data were analyzed using a broad range of size categories. Pooling of the data into under-size and legal-size categories eliminated any statistical significance (Table 6). It is also noteworthy that the temporal differences in the size structure of stripey snapper in the impact zone were not systematic through time and were largely driven by under-size categories (revealed by comparison of cell χ^2^ values). Together, these results strongly indicate that the temporal differences in the size structure of stripey snapper in the impact zone were not related to spearfishing.

### Habitat Indices

Total coral cover in the no-fishing zone declined significantly between 2004 and 2009 (Figure 3F, Table 2). This result was driven predominantly by a decline in soft coral cover, since there were no significant differences in hard coral cover during the same period (Figure 3G). Total coral cover was significantly higher in the impact zone than in the control zone (due to differences in soft coral cover), but no significant temporal differences were observed in either zone (Table 3). Similarly, structural complexity was significantly higher in the impact zone than in the control zone, but no significant temporal differences were observed in either zone (Figure 3H, Table 3). The temporal stability of total coral cover, hard coral cover and structural complexity in control and impact zones indicates that the observed changes in fish populations were independent of habitat quality.

## Discussion

Coincident with accession of spearfishing to the multi-use conservation park zone (CPZ) at Palm Island was a 54% reduction in density and a 27% reduction in mean size of coral trout, the primary target species of spearfishers on the Great Barrier Reef (GBR). Given that benthic habitat characteristics and densities of non-target fishes at Palm Island were stable through time, and that the density and mean size of coral trout remained unchanged in the nearby control zone (where spearfishing was prohibited), the decline in the coral trout population at Palm Island can be attributed to spearfishing. As such, this study provides direct evidence that spearfishing can have rapid and substantial negative effects on reef fish populations, even when moderate size- and catch-limits apply. Previously, there has been a distinct lack of empirical evidence regarding the effects of spearfishing on fish stocks, particularly for coral reef fisheries [4], [8], [11]. Because of this, the need for stringent management of spearfishing has been under-recognized in many countries throughout the tropical world, leading to increased risk of overfishing [8], [11]. In demonstrating that spearfishing can be detrimental to fish stocks, our study highlights the need for careful management of spearfishing to ensure that conservation goals are not compromised and that the harvest of fishery resources is sustainable. This is particularly important for areas with extraordinarily high conservation value such as the GBR World Heritage Area [15] and for the many developing tropical island nations where people depend on spearfishing for food and income [8], [13], [25].

Because most reef fisheries utilize multiple gears, it is pertinent to understand the effects of spearfishing relative to other common forms of fishing such as linefishing. Although previous studies indicate that spearfishing can be more efficient than linefishing in terms of catch-per-unit-effort (CPUE) of target species [5], [9], the overall impacts of spearfishing and linefishing appear broadly equivalent once collateral damage such as bait consumption, bycatch and pollution (lost gear) are considered [4]. In the present study, mean densities of coral trout in the control and impact zones in 2004 (before rezoning) were equivalent, but lower than the mean density of coral trout in the no-fishing zone (Figure 3A). The most parsimonious explanation for this result is the historical presence of linefishing in control and impact zones. Importantly, in 2007 (3 yr after rezoning), the absolute difference in mean density of coral trout between control and impact zones was similar to that between control and no-fishing zones (Figure 3A). It is plausible that post-2004 spearfishing reduced the density of coral trout in the impact zone by an amount equivalent to that of pre-2004 linefishing, and that the independent effects of spearfishing and linefishing are additive. In any case, there is no evidence to suggest that the effects of spearfishing on coral trout populations are any different to those of linefishing at current effort levels. In this respect, it would seem appropriate that input controls (e.g. spatial and temporal closures) be applied equitably across both spearfishing and linefishing sectors, at least in the GBR.

The general negative effects of fishing on reef fishes have been well established (reviewed by [26]). The consensus is that excessive fishing pressure (1) reduces the abundance, mean size and reproductive potential of target fishes and (2) pervasively alters community-level interactions that ultimately reduce ecological resilience and biodiversity [6]. The results presented here are consistent with this view, at least at the population level (no. 1). Although community-level consequences of over-exploiting coral trout are conceivable (because coral trout are high trophic-level predators: [27]), results from the present study suggest that any such effects are either slow to manifest (>5 yr) or affect elements of the reef community other than those quantified here (i.e. not coral cover, density of rabbitfish, etc.). Given that coral trout are heavily targeted throughout the GBR and elsewhere in the Indo-Pacific region [28], detailed studies of the community-level effects of exploiting these large, potentially-keystone species are an urgent priority.

Fishers typically target the larger individuals in a population, which tends to reduce the mean size of fished species [26]. At Palm Island, spearfishing reduced the mean size of coral trout by 27% during the five years from 2004 to 2009. Intense, size-selective fishing pressure is concerning to fishery managers because reproductive output declines exponentially with decreasing fish size, such that depletion of the larger individuals in a population can rapidly precipitate recruitment over-fishing [26], [29]. Another management consideration is that coral trout are protogynous hermaphrodites (change sex from female to male), so size-selective fishing can alter sex ratios and limit sperm availability via disproportionate removal of larger, male individuals [29]. Fishing selection is thought to be strongest for spearfishing because it is more size-selective than any other fishing method [3]. This must be considered in any future policy debate about the management of spearfishing.

Other studies have investigated the density and size structure of primary and secondary target fishes in relation to fishing pressure at the Palm archipelago [17], [30]. Although these studies did not survey CPZs or explicitly consider the independent effects of spearfishing, some useful inter-study comparisons are still possible. Perhaps the most striking unanimous result among studies is the higher densities (1.4–3.6 fold) and larger mean sizes (1.1–1.7 fold) of coral trout in no-fishing zones than in fished zones, despite spatial and temporal separation of survey samples by up to 30 km and 10 yr, respectively (c.f. this study and [17], [30]). The magnitude of these inter-zone differences in density and mean size confirms that coral trout are subjected to moderate to high fishing pressure at the Palm archipelago. Furthermore, the consistency of the inter-zone differences through time, space, and between human observers, provides strong empirical evidence that no-take marine reserves afford substantial conservation benefits for coral trout, even when the reserve is relatively small in size. With respect to density of stripey snapper, neither the present study nor a previous study [30] found any significant difference between no-fishing and fished zones, suggesting that stripey snapper are targeted less than coral trout and (or) that stripey snapper cope well with fishing pressure. In either case, a sensible option for fishery managers is to use the latent yield of stripey snapper to relieve the fishing pressure on coral trout, thereby reducing the risk of over-fishing and the potential effects of fishing selection on the gene pool. A shift in fishing effort among species could be easily achieved by adjusting the relative size- and (or) catch-limits for each species.

Given the critical role of herbivorous fishes in preventing competitive exclusion of coral by algae, the effects of fishing on these keystone fishes are a priority concern for reef managers [6]. Unlike linefishing, spearfishing can target herbivores such as parrotfish (Scaridae) and surgeonfish (Acanthuridae), and considerable quantities are harvested by reef fisheries worldwide [5], [13], [25]. To maximize ecological resilience of coral reefs and (or) to promote recovery of degraded reefs, Cinner et al. [5] suggested prohibition of spearfishing in favour of other fishing methods such as linefishing, thereby reducing catches of functionally important herbivores. In our study, no significant changes in the density of parrotfish were observed after accession of spearfishing to the CPZ at Palm Island, presumably because local catch rates of parrotfish were very low [4]. To maintain *status quo* and ensure that parrotfish populations continue to fulfil their essential ecosystem function, we recommend that fishery managers proactively enhance protection of parrotfish and other herbivores before any shift in the composition of spearfishers’ catches. Because spearfishing has substantial cultural and social importance in many coastal communities in the region [4], [10], we suggest that the most appropriate and politically acceptable way to protect herbivorous fishes is to strengthen output controls such as size-and catch-limits rather than prohibit spearfishing altogether (as per [5]).

To deduce the effects of spearfishing on fish populations, we assumed that (1) spearfishers accessed the CPZ after it was opened to spearfishing on 1 July 2004, and (2) the distribution of linefishing effort remained unchanged after 1 July 2004. Although the validity of these assumptions is strongly supported by multiple lines of evidence (see Materials and Methods section), we did not quantify spearfishing or linefishing pressure (catch or effort) because it was considered beyond the scope of this study due to the dispersed nature of the fishery and the lack of reporting requirements for local fishers [4]. Thus, we are unable to infer the fishing pressure that caused coral trout populations to decline or the level of fishing pressure that is sustainable for the CPZ at Palm Island. However, by demonstrating the ecological effects of spearfishing at the current level of fishing effort, this study provides a solid justification for future studies that aim to quantify catch and effort of this fishery.

In summary, we conclude that spearfishing, like other forms of reef fishing, can have rapid and substantial negative effects on the density and mean size of target fish populations, even when moderate size- and catch-limits apply. As such, this study highlights the need for careful management of spearfishing to ensure that conservation obligations are achieved and that fishery resources are harvested sustainably. This is particularly important both for the GBR, due to its extraordinarily high conservation value and world heritage status, and for tropical island nations where people depend on spearfishing for food and income. Lastly, we recommend that fishery managers adjust output controls to preserve critical ecosystem functions (herbivory) and to balance exploitation rates between primary and secondary target species (e.g. coral trout versus stripey snapper). These management actions will help to maximize ecological resilience of coral reefs and minimize the effects of spearfishing on exploited species, whilst allowing the continuation of a culturally and socially important activity.
